# Reproductive justice for incarcerated mothers and advocacy for their infants and young children

**DOI:** 10.1002/imhj.21810

**Published:** 2019-07-19

**Authors:** Rebecca J. Shlafer, Rachel R. Hardeman, Elizabeth A. Carlson

**Affiliations:** ^1^ Department of Pediatrics University of Minnesota Minneapolis Minnesota; ^2^ School of Public Health University of Minnesota Minneapolis Minnesota; ^3^ Institute of Child Development University of Minnesota Minneapolis Minnesota

**Keywords:** incarcerated mothers, infant mental health, pregnant incarcerated women, young children, madres encarceladas, mujeres embarazadas encarceladas, niños pequeños, salud mental infantil, mères incarcérées, mères incarcérées enceintes, jeunes enfants, santé mentale du nourrisson, inhaftierte Mütter, schwangere inhaftierte Frauen, kleine Kinder, psychische Gesundheit von Kindern, 収監された母親, 収監された妊婦, 幼児, 乳幼児のメンタルヘルス, 被監禁的母親, 被監禁的懷孕婦女, 幼兒, 嬰兒的心理健康, الأمهات السجينات, الحوامل السجينات, الأطفال الصغار, الصحة النفسية للرضع

## Abstract

The United States has seen unprecedented growth in the number of incarcerated women, most of whom are mothers with minor children. Major public health concerns relate to the reproductive health of women in prisons and jails and the well‐being of their infants and young children. In the current article, we use a reproductive justice framework to examine the intersection of incarceration and maternal and child health. We review (a) current research on the reproductive health of incarcerated women, (b) characteristics and experiences of pregnant incarcerated women, (c) outcomes of infants and young children with incarcerated parents, (d) implications of research findings for policy and practice, and (e) the need for increased research, public education, and advocacy. We strongly recommend that correctional policies and practices be updated to address the common misconceptions and biases as well as the unique vulnerabilities and health needs of incarcerated women and their young children.

## RESUMEN

Estados Unidos ha visto un crecimiento sin precedentes en el número de mujeres encarceladas, la mayoría de las cuales son madres con niños menores. Mayores preocupaciones de salud pública conciernen a la salud reproductiva de mujeres en prisiones y cárceles y el bienestar de sus infantes y niños pequeños. En el presente artículo, usamos un marco de trabajo de justicia reproductiva para examinar la intersección del encarcelamiento y la salud materno‐infantil. Revisamos 1) la investigación actual sobre la salud reproductiva de mujeres encarceladas, 2) las características y experiencias de mujeres embarazadas encarceladas, 3) lo que resulta de infantes y niños pequeños con progenitores encarcelados, 4) las implicaciones de los resultados de la investigación en cuanto a políticas y prácticas, y 5) la necesidad de aumentar la investigación, la educación pública y la defensoría. Recomendamos fuertemente que se actualicen las políticas y prácticas correccionales para incluir los conceptos erróneos y prejuicios acerca de mujeres encarceladas y sus niños pequeños, así como también sus distintivas vulnerabilidades y necesidades de salud.

## RÉSUMÉ

Les Etats‐Unis d'Amérique ont été les témoins d'une augmentation sans précédent dans le nombre de femmes incarcérées, la plupart étant des mères avec des enfants mineurs. De grands problèmes de santé publique sont liés à la santé reproductive des femmes en prison et au bien‐être de leurs nourrissons et de leurs jeunes enfants. Dans cet article nous utilisons une structure de justice reproductive afin d'examiner l'intersection de l'incarcération et de la santé maternelle et de l'enfant. Nous passons en revue: 1) les recherches actuelles sur la santé reproductive des femmes incarcérées, 2) les caractéristiques et les expériences de femmes incarcérées enceintes, 3) les résultats de nourrissons et de jeunes enfants avec des parents incarcérés, 4) les implications des résulats de recherche pour les lois et la pratique, et 5) le besoin de plus de recherches, plus d’éducation publique, plus de promotion et défense. Nous recommandons fortement que les lois correctionnelles et les pratiques correctionnelles soient mises à jour afin de traiter et de faire face aux conceptions erronées et à la partialité communes, ainsi qu'aux vulnérabilités uniques et aux besoins de santé des femmes incarcérées et de leurs jeunes enfants.

## ZUSAMMENFASSUNG

In den Vereinigten Staaten ist die Zahl der inhaftierten Frauen, von denen die meisten Mütter mit minderjährigen Kindern sind, beispiellos gestiegen. Wesentliche Bedenken der öffentlichen Gesundheit betreffen die reproduktive Gesundheit von Frauen in Gefängnissen sowie das Wohlergehen ihrer Säuglinge und Kleinkinder. Im vorliegenden Artikel verwenden wir ein Rahmenkonzept der reproduktiven Gerechtigkeit, um den Schnittpunkt von Inhaftierung und Gesundheit der Mütter und Kinder zu untersuchen. Wir betrachten 1) die aktuelle Forschung zur reproduktiven Gesundheit von inhaftierten Frauen, 2) Merkmale und Erfahrungen von schwangeren inhaftierten Frauen, 3) Ergebnisse von Säuglingen und Kleinkindern inhaftierter Eltern, 4) Implikationen von Forschungsergebnissen für Politik und Praxis und 5) den höheren Bedarf an Forschung, öffentlicher Bildung und Interessenvertretung. Wir empfehlen nachdrücklich, die Korrekturmaßnahmen und ‐praktiken zu aktualisieren, um den häufigen Missverständnissen und Verzerrungen sowie den spezifischen Vulnerabilitäten und Gesundheitsbedürfnissen von inhaftierten Frauen und ihren kleinen Kindern angemessen zu begegnen.

## 抄録

収監された母親の生殖の権利と乳幼児の擁護活動

合衆国では収監された女性の数がかつてないほど増加しており、そのほとんどは少人数の子どもを持つ母親である。公衆衛生の主要な関心事は、刑務所や拘置所に収監されている女性の生殖に関連する健康とその乳幼児の福祉に関することである。われわれは、この論文で生殖の権利の枠組みを用いて、収監と母親や子どもの健康に共通する部分について検討した。われわれは、1) 収監された女性の生殖に関連する健康についての現在の研究、2) 収監された妊娠女性の特徴と経験、3) 収監された両親を持つ乳幼児の転帰、4) 政策や取り組みのための研究成果の重要性、そして5) さらなる研究、学校教育、そして擁護活動の必要性、について再検討した。われわれは、収監された女性とその乳幼児の持つ特有の脆弱性と健康の必要性のみならず、よくある誤解や偏見に対処するためにも、懲治政策や取り組みを更新すべきであることを強く推奨する。

## 摘要

監禁母親的生殖公義及其嬰兒和幼兒的支援

美國被監禁婦女的人數出現前所未有的增長, 其中大多數是有未成年子女的母親。主要的公共衛生問題涉及監獄和拘留所中婦女的生殖健康及其嬰兒和幼兒的福祉。在本文中, 我們使用生殖公義框架來查考監禁與母嬰健康的交叉點。我們回顧 1) 目前關於被監禁婦女生殖健康的研究, 2) 被監禁的懷孕婦女的特徵和經歷, 3) 被監禁父母的嬰兒和幼兒的結果, 4) 研究結果對政策和實踐的影響, 以及 5) 需要加強研究丶公眾教育和宣傳。我們強烈建議更新懲教政策和做法, 以解決其他人對被監禁婦女及其幼童常見的誤解和偏見, 以及理解他們獨特的脆弱性和健康需求。

## ملخص

**العدالة الإنجابية للأمهات السجينات والدفاع عن أطفالهن الرضع وأولادهن الصغار **

شهدت الولايات المتحدة نموا لم يسبق له مثيل في عدد السجينات، ومعظمهن أمهات لديهن أطفال قاصرون. وتتمثل الشواغل الرئيسية في مجال الصحة العامة بالصحة الإنجابية للنساء في السجون ورفاهية أطفالهن الرضع والأطفال الصغار. وفي هذه المقالة، نستخدم إطارا للعدالة الإنجابية لدراسة التقاطع بين السجن وصحة الأم والطفل. نستعرض 1) البحوث الحالية حول الصحة الإنجابية للسجينات، 2) خصائص وتجارب السجينات الحوامل، 3) نتائج الرضع والأطفال الصغار مع الآباء المسجونين، 4) تطبيقات نتائج البحوث في السياسة والممارسة، و 5) الحاجة إلى زيادة البحوث، والتثقيف العام، والدعوة. ونوصي بشدة بتحديث السياسات والممارسات الإصلاحية لمعالجة المفاهيم الخاطئة والتحيزات الشائعة، فضلا عن أوجه الضعف والاحتياجات الصحية الخاصة للسجينات وأطفالهن الصغار.

## 1 INTRODUCTION

Over the last three decades, the United States has seen unprecedented growth in the number of women involved in the criminal justice system; the number of women incarcerated in the United States has increased by more than 700% (Bureau of Justice Statistics, 1986, 2014, 2015; Carson, 2018). This growth has largely been attributed to changes in state and federal policies, including harsher drug‐sentencing laws that have had a dramatic impact on women in particular. A growing body of evidence has pointed to gender‐specific differences that are driving growth in the female prison population, including women's complex histories of abuse and trauma, mental illness, barriers to seeking and obtaining effective gender‐specific treatment for mental health and substance‐abuse problems, patterns of drug use, and women's involvement in the drug trade (American Civil Liberties Union [ACLU], 2005).

Changes to state and federal policy have disproportionately impacted women of color and women from low‐income communities (ACLU, 2005; The Sentencing Project, 2015). In 2014, the imprisonment rate was 109 per 100,000 for Black women, compared to 64 per 100,000 Hispanic women, and 53 per 100,000 for White women (The Sentencing Project, 2015; Data for American Indian women were not included in this report.) These racial disparities are driven by systemic inequities in our society which disproportionately place women of color in contact with the criminal justice system (Sufrin, Kolbi‐Molinas, & Roth, 2015). Systemic inequities embedded within the criminal justice system, including (but not limited to) racially targeted law enforcement, disproportionate access to legal representation, and prosecutorial discretion, disproportionately impact low‐income women and women of color (ACLU, 2005).

The dramatic increase in the number of incarcerated women has had devastating consequences for women and their children, families, and communities. Although there are substantially more fathers than mothers incarcerated in the United States, maternal incarceration is increasing at a much faster rate (Maruschak, Glaze, & Mumola, 2010). Between 1991 and 2007, the number of fathers in prison increased 77% whereas the number of mothers in prison increased by 122% during the same time period (Maruschak et al., 2010). Most (76%) incarcerated women are of childbearing age (18–44 years old; Carson & Sabol, 2012). A majority (61%) report having minor children (Maruschak et al., 2010), and an estimated 3 to 4% of women in prison are pregnant at the time of their admission (Maruschak, 2008). Major public health concerns relate not only to the reproductive health of incarcerated women but also to the health and well‐being of their infants and young children.

In the current article, we examine the intersection of incarceration and maternal and child health, using the core principles of reproductive justice (Ross, 2006; Sufrin et al., 2015). Reproductive justice combines reproductive rights and principles of social justice and is grounded in the internationally‐accepted human rights framework created by the United Nations in 1948 (United Nations, 2019; SisterSong, 2019). The framework is the creation of a group of Black women (SisterSong) who, in 1994, grew weary of the women's rights movement led by and representative of middle and upper class White women (Ross, 2006). As defined by SisterSong and outlined by Derr (2017), reproductive justice is “the human right of every person to: 1) decide if and when she will have a baby and the conditions under which she will give birth; 2) decide if she will not have the baby and her options for preventing or ending a pregnancy; and 3) parent her children with the necessary social supports in safe environments and healthy communities and without fear of violence from individuals or the government” (p. 88). Using this framework, we provide an overview of the current research and consider the implications of incarceration for the health of women and the well‐being of their infants and young children.

### 1.1 Parental incarceration: Jail or prison

“Parental incarceration” is often used as an umbrella term, referring to the incarceration of a child's mother or father in jail or prison; however, distinguishable differences in facility types have important implications for parents and their young children. Jails are locally operated correctional facilities that confine persons before or after adjudication (the judicial decision or sentence). Sentences to jail (typically misdemeanors or gross misdemeanors) are usually 1 year or less whereas sentences to prison (typically felonies) are generally more than 1 year (BJS, 2010). Although there may be similarities between individuals incarcerated in both jails and prisons (e.g., history of substance use, mental health problems), there are generally important differences in the offense type, sentence length, and availability of services. In general, prisons house individuals with more serious offenses, for longer periods of time, and often have the space, infrastructure, and staff to provide additional services (e.g., remedial education, chemical health treatment, parenting education), as compared to local/county correctional facilities. Notably, though, nearly everyone who is sentenced to prison has been confined to a jail pre‐adjudication, making the jail population highly diverse in terms of sociodemographic background, criminal history, and criminal charges.

The location of correctional facilities and its related visitation policies have important implications for children and families. Compared to prisons, jails are often closer to an incarcerated person's residence at the time of arrest, potentially impacting the frequency of family visits (Shlafer, Loper, & Schillmoeller, 2015). In addition, visits in jails are often noncontact, meaning that the incarcerated person and their visitors are separated by Plexiglas and communicate through a telephone or through closed‐circuit television (i.e., video visitation). In contrast, prisons frequently provide incarcerated people with the opportunity to interact directly and, in some cases, hold their young children.

Because there are fewer women in prison than men, states often have only one or two facilities that house women. As a result, women's prisons tend to be located substantially farther from their homes than are men's prisons (Eddy & Poehlmann, 2010; Shlafer, Loper, & Schillmoeller, 2015). These differences in correctional settings are important to keep in mind when considering the impact of incarceration on mothers and their children, particularly in light of a reproductive justice framework. Indeed, the location, distance, access, safety, and availability of resources and supports within a correctional facility directly relate to an incarcerated mother's ability to “parent her children with the necessary social supports in safe environments and healthy communities” (Derr, 2017, p. 88). We will revisit these issues again in our discussion of the effects of maternal incarceration on child outcomes.

### 1.2 Characteristics of incarcerated mothers

In this section, we briefly describe demographic and social factors associated with maternal incarceration, including gender, racial and ethnic background, education, economics, and history of trauma and adversity. Note that these factors are associated with conditions of disadvantage, increasing one's risk for initial involvement with the criminal justice system, with implications for child and family well‐being.

First, incarcerated women are more likely to report having minor children than are incarcerated men, and more mothers than fathers report living with their minor children in the month prior to arrest (Glaze & Maruschak, 2008). Although the likelihood of being a mother does not vary by race (i.e., White women in prison are just as likely to report being mothers as are women of color), the racial disparities in incarceration rates result in children from racial and ethnic minority backgrounds being disproportionately impacted by their mothers’ incarcerations (Glaze & Maruschak, 2008). Using data from the National Survey of Children's Health, Murphey and Cooper (2015) reported that Black children are nearly twice as likely to experience the incarceration of a parent they lived with than are White children (11.5 vs. 6%, respectively). Proposed explanations for these racial disparities range from variations in offending based on race, biased policing, and decision‐making in the criminal justice system as well as a wide range of social factors such as poverty, education, and unemployment (Garland, Spohn, & Wodahl, 2008). Systemic racist or biased policies that advantage certain populations while disadvantaging others also may contribute to disparities. For example, with the escalation of the “war on drugs” in the 1980s, the United States also experienced a dramatic increase in incarceration of women of color (Mauer, Potler, & Wolf, 1999).

Low educational attainment and economic instability are common characteristics among the parents in prison. Among incarcerated mothers in state prisons who reported having lived with their minor children, 35% had not graduated from high school or received their General Educational Diploma (GED) at the time they entered prison (Glaze & Maruschak, 2008). Based on Bureau of Justice Reports (Glaze & Maruschak, 2008), incarcerated mothers report considerable economic risk—more than one third of mothers in state prison reported receiving government transfers (e.g., welfare, social security), and compared to fathers in state prison, mothers are nearly twice as likely to report homelessness in the year prior to their arrest.

Many incarcerated parents have had difficult childhoods. Compared to incarcerated fathers, incarcerated mothers report more adversity and trauma (Glaze & Maruschak, 2008). For example, approximately 16% of incarcerated fathers, but nearly 60% of incarcerated mothers, reported a history of physical or sexual abuse (Glaze & Maruschak, 2008). Beyond abuse, other adverse childhood experiences (ACEs) are common among incarcerated women (De Ravello, Abeita, & Brown, 2008; Friestad, Åse‐Bente, & Kjelsberg, 2014; Messina & Grella, 2006). Just less than half (41%) of mothers in state prison report ever receiving public assistance; 17% report ever living in a foster home, agency, or institution; and 43% report that a parent or guardian had abused drugs or alcohol (Glaze & Maruschak, 2008).

In addition to (or as a result of) ACEs, many incarcerated parents report physical, chemical, and mental health issues that may have interfered with their parenting capacity pre‐incarceration. These challenges are more commonly reported among mothers than fathers. For example, from a state prison report, 53% of mothers reported a current medical problem (vs. 40% of fathers), nearly 74% of mothers met criteria for a mental health problem, and 7 in 10 met criteria for substance dependence/abuse (Maruschak et al., 2010).

Thus, parental incarceration, particularly maternal incarceration, is frequently associated with co‐occurring risk factors such as a history of adversity and trauma, poverty, single parenthood, limited education and employment, parent substance use, and mental and physical health problems. Many of the same factors that place mothers at risk for involvement in the criminal justice system are also risks known to compromise effective parenting and child well‐being (Zeanah, 2009).

### 1.3 Characteristics of pregnant incarcerated women

Research has indicated that incarcerated women who are pregnant face additional risks, even beyond the sociodemographic and health risks described earlier. When compared to women in the general population, incarcerated pregnant women are more likely to experience risk factors associated with poor maternal and infant health, including preterm and small‐for‐gestational‐age infants (Bell et al., 2004; Knight & Plugge, 2005). The outcomes are likely a result of multiple factors that may precede incarceration (Kotlar et al., 2015), including substance use (Knight & Plugge, 2005), chronic medical conditions (Knight & Plugge, 2005), stress and depressive symptoms (Hutchinson, Moore, Propper, & Mariaskin, 2008), violence exposure (DeHart, Lynch, Belknap, Dass‐Brailsford, & Green, 2014), poor nutrition (Ferszt & Clarke, 2012), sexually transmitted infections (Clarke et al., 2006), and limited access to reproductive care (Clarke et al., 2006).

Given these complex risks, incarceration may offer some level of protection to the woman and her developing fetus, particularly in terms of stable housing, access to healthcare services, access to regular meals, and protection from substance use and domestic violence (Martin, Rieger, Kupper, Meyer, & Qaqish, 1997; Sufrin, 2017). Consistent with this idea, Martin et al. (1997) found that longer periods of incarceration were associated with better birth outcomes in terms of gestational age and birth weight. Yet, incarceration is an ethically complicated intervention for pregnant women, and as we describe next, correctional facilities are rarely equipped to address women's complex medical and psychosocial needs.

Notably, Black, American Indian, and Hispanic women, at highest risk for poor pregnancy and birth outcomes (Dominguez, 2008; Lu & Halfon, 2003), are also disproportionally represented in the criminal justice system (Glaze & Maruschak, 2008; Hamilton, Hoyert, Martin, Strobino, & Guyer, 2013; MacDorman, 2011). Thus, for women of color and American Indian women who are not incarcerated, risks of experiencing maternal mortality or morbidity (Flanders‐Stepans, 2000), experiencing preterm labor and delivery (Culhane & Goldenberg, 2011), and giving birth to a low‐birth‐weight or small‐for‐gestational‐age baby (Lu & Halfron, 2003; Grady, 2006) are significantly higher. Coupled with the additional risks associated with incarceration, there is great need for concern regarding this population.

While the causes of disparities in adverse birth outcomes for women of color are likely multifactorial and remain unexplained, current research with women who are not incarcerated has pointed to structural racism as a fundamental (root) cause of inequitable birth outcomes (Phelan & Link, 2015; Wallace, Mendola, Liu, & Grantz, 2015). For example, a recent study with women who were not incarcerated has found that indicators of structural racism were associated with higher odds of a small‐for‐gestational‐age birth (Wallace et al., 2015). Residential redlining as a form of structural racism may also contribute to disparities in birth outcomes (Mendez, Hogan, & Culhane, 2014). Maternal stress resulting from experiences of racism and discrimination is posited as a mechanism of transmission to birth outcomes. Indeed, the few studies of Black pregnant women have confirmed maternal stress as a risk factor in this group (vs. White women) for low birth weight and preterm delivery (Giscombé & Lobel, 2005). Additional research examining the impacts of structural racism for women in prison and other particularly marginalized groups is a valuable area for future inquiry.

### 1.4 Experiences of pregnant incarcerated women

Because many correctional policies were created when female incarceration was relatively rare, institutional policies and procedures reflect designs for incarcerated men and, in general, fail to address unique health needs and services related to the reproductive health of incarcerated women (Clarke et al., 2006; Ferszt & Clarke, 2012; Wilper et al., 2009). The current conditions of correctional facilities and the experiences of women incarcerated in these facilities are directly tied to Ross’ (2006) definition of reproductive justice, particularly the rights to decide if and when to have a baby, the conditions under which to give birth, and options to prevent or end a pregnancy. In the next section, we review some of the key issues associated with pregnancy in the context of incarceration and discuss the experiences of incarcerated pregnant women within a reproductive justice framework.

#### 1.4.1 Pregnancy‐related care

Within a reproductive justice framework (Derr, 2017; Ross, 2006), a central component of a person's right to decide whether to have a baby and the conditions under which that decision will be carried out, is ensuring that one has all the information necessary to make informed decisions—beginning with the ability to determine if one is pregnant. In a recent study by Kelsey, Medel, Mullins, Dallaire, and Forestell (2017), most (62%) jails reported that they did not provide pregnancy tests to women upon their admission to the correctional facility. Further, upon confirmation of pregnancy, fewer than 30% of the facilities reported that they provided women who are pregnant and incarcerated with information about rights to have their baby adopted or terminate their pregnancy.

For women who plan to retain custody of their infants (even if physically separated for a period of time due to the incarceration), few correctional facilities provide women with access to counseling or practical assistance (e.g., legal advice, copies of legal documents) to grant short‐term guardianship to a relative or friend. Furthermore, women may encounter significant barriers in accessing information about programs and services to support their children and their families (e.g., alternatives to incarceration, residential family‐based treatment, nursey programs). Such policies and practices fail to promote reproductive justice and contribute to systemic inequities among populations disproportionately affected by the criminal justice system. Promoting reproductive justice requires that corrections administrators and staff provide incarcerated pregnant women with information and support in making informed decisions.

Kelsey et al.’s (2017) findings are consistent with previous research documenting poor or inadequate pregnancy‐related care for incarcerated women. In a national survey of adults in prison, Maruschak (2008) found that only about half (54%) of pregnant women in state prisons received some type of pregnancy care. Furthermore, in their study of the healthcare practices of pregnant women in 19 state prisons, Ferszt and Clarke (2012) described living conditions, healthcare, and counseling practices that failed to meet women's basic needs. For example, they found that some state prisons surveyed failed to meet the nutritional recommendations for pregnant women or accommodate recommendations regarding labor, rest, sleep, and clothing needs of pregnant women. Further, a minority of the surveyed prisons provided psychosocial support services or educational programming for pregnant women. Poor maternal healthcare related to pregnancy and during incarceration not only compromises the health of women but also may have long‐term implications for their offspring (Dumont et al., 2014; Lu & Halfon, 2003), violating the third principle of the reproductive justice framework—the right to raise children with dignity and safety (Derr, 2017; Ross, 2006).

Differences in type of correctional facility have important implications for incarcerated pregnant women. For those who are pretrial and have the financial resources to post bail, stays may be very short (i.e., hours or days) and have limited impact on a woman depending on her health history, the health of her fetus, and her access to health services prior to confinement. However, for women with high‐risk pregnancies, even short stays can have devastating consequences. For opioid‐dependent pregnant women, for example, a short jail stay with unsafe detox and withdrawal protocols could result in fetal death. Indeed, Kelsey et al. (2017) found that nearly half (46%) of jails put opioid‐addicted pregnant women through withdrawal protocols, despite clinical best practices which advise against doing so.

#### 1.4.2 Childbirth

Because jail sentences are often for lower level offenses with shorter periods of incarceration, pregnant women serving sentences in local jails are often released before the birth. In many instances, judges may grant furloughs so that jailed women can be released into the community to give birth, and in some instances, judges may reduce a woman's sentence so that she does not have to be separated from her infant after birth. In other instances, a judge may request that a mother complete her sentence at a later date. Little is known about the prosecutorial and judicial discretion that is used for pregnant incarcerated women in these circumstances and whether disparities exist based on race or other biases. However, current practices appear inconsistent with a reproductive justice framework that would promote the right to determine the conditions under which a mother would have a baby.

The experience for pregnant women in prison often differs significantly from that of pregnant women in jail. Because sentences to prison are generally more than 1 year, a woman who enters prison pregnant will most often give birth during her incarceration. Although published studies on this phenomenon are rare, the childbirth experiences of incarcerated women have been described as poor. Schroeder and Bell (2005) described their observations of the typical labor and delivery experiences of incarcerated women:

Incarcerated pregnant women were typically transferred to a local hospital in early labor to forestall birth in jail, often arriving in leg irons or handcuffs. Once admitted, they were not permitted to leave the hospital room, have visitors, or use the phone. Labor and birth routinely took place in the presence of multiple unfamiliar providers, under constant sight surveillance of armed officers. After birth, mothers were usually transferred back to the jail within 24 hours, while their babies were placed with a relative or foster family supervised by child welfare services. Often, infant placement could not be arranged before birth, adding to the mothers’ anxiety. (p. 53)

While possibly outdated, these observations are generally consistent with our work with imprisoned women in Minnesota (Shlafer, Gerrity, & Duwe, 2015) and our understanding of the processes and policies in other states (for a review, see Goshin, Arditti, Dallaire, Shlafer, & Hollihan, 2017). Indeed, Kelsey et al. (2017) reported that 17.4% of facilities surveyed require incarcerated women to be handcuffed or shackled during the delivery process. Shackling has been widely criticized for the potential health risks to pregnant women and their fetuses, and numerous professional associations have expressed opposition to these policies (Goshin et al., 2017).

Finally, we wish to acknowledge that although most women are in prison for nonviolent offenses (Carson, 2018), some incarcerated women have committed very serious and violent offenses, or have serious and persistent mental health issues, that may pose a threat to themselves or others. Such concerns regarding the woman or the public's safety may severely constrain her rights under a reproductive justice framework to determine the conditions under which she gives birth.

#### 1.4.3 Postpartum experiences

Women who give birth while still under correctional custody are typically separated from their newborns within 48 to 72 hr after delivery (limiting opportunities for parent–infant bonding, initiation of breast‐feeding, and other nurturing maternal practices). Even the short period of time that women have with their newborns in the hospital is limited, as shackling is also common postpartum. In their study, Kelsey et al. (2017) found that most (56.5%) facilities reported restraining women in the postpartum period.

For mothers with longer sentences, principal concerns turn to where their infants will reside following the mother–child separation. The concern is directly related to the third principle of reproductive justice; namely, the right to “parent children with the necessary social supports in safe environments and healthy communities and without fear from violence from individuals or the government” (Derr, 2017, p. 88). National data on placement of infants born to incarcerated women—or their outcomes following the mother's release from prison—are not available. However, Schroeder and Bell's (2005) descriptions are consistent with our observations of incarcerated pregnant women in Minnesota's state prison. In Minnesota, pregnant women work with the prison's parenting coordinator to identify a placement resource for the infant. Most often, a maternal relative (e.g., the infant's grandmother, aunt) picks up the baby from the hospital and takes temporary custody in anticipation of the mother returning to a primary caregiving role upon release from prison. As discussed earlier, few resources are available to assist these mothers with understanding the legal implications of such informal placements.

In Minnesota, approximately one third of infants born to incarcerated mothers live with their biological fathers following hospital discharge (L. Timlin, personal communication, October 7, 2015). For many women, these family arrangements are often complex. Although many women report feeling thankful that a family member was willing to provide care for their baby or that the baby would live with his or her father, mothers may simultaneously have concerns about negative childhood experiences being repeated (e.g., physical abuse) or intergenerational patterns of substance abuse and/or mental illness.

Challenges with coparenting (e.g., history of domestic violence, conflict) and barriers to visitation (e.g., the cost of travel, distance to the facility) may mean that mothers have limited or no contact with their infants until their release from prison. To date, no studies have systematically examined the caregiving arrangements of infants born to incarcerated women, the capability or stability of these caregivers, how incarcerated mothers and caregivers coparent during incarceration, or how they navigate coparenting and reunification upon the mother's release. These are valuable areas for future inquiry and intervention.

In instances in which child protection is involved and the baby is placed in foster care, incarcerated mothers face additional challenges. Prison sentences are often incompatible with child protection timelines, in that prison sentences may exceed state and federal statutes specifying allowable duration of out‐of‐home placement for young children. In addition, incarcerated mothers may have limited opportunities (e.g., substance‐abuse treatment, domestic‐abuse counseling) to work a case plan toward reunification.

Prison nursery programs offer an alternative to separating newborns from their incarcerated mothers. Such programs are only available in eight states and one jail (Riker's Island in New York City) and allow infants to co‐reside with their mothers in the correctional facility, facilitating maternal physical and emotional nurturance (Goshin et al., 2017). The programs also vary considerably in the number of mother–infant dyads served, the length of infant stay, and the eligibility criteria for incarcerated mothers (for a review, see Goshin et al., 2017). To date, the most rigorous research on prison nursery programs has been conducted at Bedford Hills Correctional Facility in New York. Byrne (2010) described positive developmental and behavioral outcomes for infants and young children who participated in the prison nursery program at Bedford Hills.

### 1.5 Effects of parental incarceration on infants and young children

In this section, we consider the effects of parental incarceration on infants and young children. As the literature on the developmental outcomes of infants born to incarcerated women is limited, we broaden our discussion to consider what is known about outcomes among infants and young children who experience parental incarceration more generally, and when available, review information specific to maternal incarceration.

Parental incarceration has been viewed as an ACE linked to physical, social, emotional, and educational outcomes across the life course (Felitti et al., 2019; Shonkoff et al., 2012). Notably, children under the age of 6 years who have experienced the incarceration of a coresident parent have, on average, 1.6 more other ACEs than do children who do not experience this ACE (Murphey & Cooper, 2015). Children (of all ages) with incarcerated parents are at increased risk for negative outcomes such as social, emotional, and behavioral disorders, delinquency, and substance use as well as cognitive delays and academic challenges (for a review, see Eddy & Poehlmann, 2010). Infants and young children of incarcerated mothers are at particular risk for attachment disturbances related to separation from a primary caregiver and inconsistent, insensitive, or unresponsive care during the mother's incarceration (Poehlmann, 2010). These early caregiver–child relationships have important consequences for subsequent health and development.

A growing body of evidence has demonstrated that the impact of parental incarceration on infants and young children likely depends on a number of factors, including the child and families’ experiences prior to, during, and after the parent's incarceration. Indeed, some scholars have argued that each family's circumstances are so different and complex that generalizing “children with incarcerated parents” oversimplifies and potentially misrepresents the complexities of these families (Genty, 2012). For example, we know little about how experiences of maternal incarceration differ for infants, toddlers, and preschoolers (as well as older children) with differing cognitive and emotional regulatory capacities, or how child outcomes may be related to circumstances of maternal incarceration. The following sections address factors that influence infant and early childhood adjustment before, during, and after a parent is incarcerated, and consider the third principle of reproductive justice; namely, the right to “parent children with the necessary social supports in safe environments and healthy communities and without fear from violence from individuals or the government” (Derr, 2017, p. 88).

### 1.6 Before incarceration

Three fourths of mothers (vs. one fourth of fathers) have reported providing primary care for their children prior to arrest (Maruschak et al., 2010). As a result, children of incarcerated mothers may be more likely to experience significant disruptions in their caregiving environments, placing young children at higher risk for attachment disturbance and compromising trajectories of health, development, and learning (Dallaire, 2007; Murray & Murray, 2010; Poehlmann, 2010; Sroufe, Egeland, Carlson, & Collins, 2005).

Prior to parental incarceration, potentially traumatic events related to the incarceration and separation from the parent as well as preexisting contextual risks (e.g., poverty, low maternal educational attainment, racism, etc.) pose challenges for healthy development and the well‐being of infants and young children. Incarceration‐related events (e.g., Dallaire & Wilson, 2010) may include witnessing the parent's criminal activity (e.g., being present for drug deals), arrest, and/or trial and sentencing. Children may also be the victims of their parent's criminal activity (e.g., physical or sexual abuse) or be exposed to another family member being victimized (e.g., domestic violence). In particular, children with incarcerated mothers are more likely than are children with incarcerated fathers to have been exposed to their parent's criminal activity, arrest, and sentencing (Dallaire & Wilson, 2010). Such experiences may have a profound impact on a child's sense of safety and security, compromising social, emotional, and cognitive development (Dallaire & Wilson, 2010). Events such as witnessing a parent's arrest (i.e., seeing the police arrive, likely without warning and often with weapons drawn), watching the parent be handcuffed and arrested, and being separated when the parent is driven away in a police car are often emotionally charged events and may be confusing or frightening for young children, especially in the absence of alternative caregivers or supportive professionals (Schechter & Willheim, 2009).

Infants and young children lack the cognitive, linguistic, and emotional capacities to fully understand and process the facts or circumstances related to a parent's criminal activity, arrest, or incarceration as well as to express their own feelings and concerns (Poehlmann, 2010). For example, young children are also likely to express concerns or distress related to the absence of the primary caregiver and the sights and sounds surrounding the sequence of events in behavior and physiological symptoms versus language (Dallaire & Aaron, 2010; Schechter & Willheim, 2009). Such behavioral and physiological responses to extreme experiences may be misinterpreted by adults (e.g., eliciting discipline) rather than linked to experiences of traumatic stress. A stable, consistent alternative caregiving environment, as well as early childhood mental health consultation, may help to buffer and interpret these responses (discussed later).

### 1.7 During incarceration

#### 1.7.1 The child's living arrangement

Because most incarcerated mothers report living with their minor child prior to arrest (Maruschak et al., 2010), the mother's incarceration may result in an infant or young child's (often sudden) separation from a primary caregiver during a formative developmental period, with significant negative consequences for later functioning. Frequently, when fathers are incarcerated, children remain with their mothers (Glaze & Maruschak, 2008) and may experience minimal disruption in the home environment or family system (in part, because the father may not have been living in the home or providing routine care for the child). In contrast, when mothers are incarcerated, the majority of children live with a grandparent or other family members (Glaze & Maruschak, 2008). In some instances, grandparents may have served as the primary caregiver or have had an active role in providing care for the child before the parent's incarceration (e.g., when a parent's chemical or mental health problems interfered with the capacity to parent); in other instances, grandparents may be taking on new roles as primary caregivers. However, for infants and young children, for whom primary caregivers are not easily interchangeable, unpredictable major separations or disruptions and transition in care pose risks for the formation and stability of foundational relationships (Rubin, Springer, Ziotnik, Kang‐Yi, & the Council on Foster Care, Adoption, and Kinship Care, 2017).

Developmental science has provided strong evidence that early experiences profoundly impact rapidly developing systems in children between birth and 3 years of age. For infants and toddlers, stable, consistent responsive interactions with an adult caregiver provide the foundation for healthy brain development (Lupien, McEwen, Gunnar, & Heim, 2009; Shonkoff et al., 2012). Through the course of routine care, the caregiver learns to read, interpret, and respond to infant signals and communications, including signs of distress (Sroufe, 1996). While older children are capable of communicating needs, understanding simple narrative explanations, and maintaining relationships to some degree through language, infants and toddlers depend on caregivers to learn and respond to a range of signals and behaviors to address their basic survival requirements (e.g., eating, staying dry and warm, human interaction and contact). Based on a history of caregiver‐orchestrated interactions, young children also increasingly organize behavior, regulate emotion, and develop expectations in relation to the caregiver. These core regulatory patterns guide subsequent development (Sroufe et al., 2005).

Caregiver absence and markedly discrepant or inconsistent caregiving have profound effects on early developmental processes. Significant separations from primary caregivers disrupt early biological rhythms and dyadic regulatory processes as well as child efforts to explore and develop an autonomous sense of self. Without consistent support, infant and toddler responses to disruption and distress may be manifested in inconsolable crying, tantrum behavior, eating and sleeping difficulties, clinging behavior, withdrawal, irritability, aggression, and/or physical symptoms and diagnoses that may require intervention. Alternative caregivers serve critical roles in providing nurturance and maintaining stability and consistency in the home environment (through the maintenance of family structures, routines, and healthy practices) to minimize disruption. In addition to caregiving sensitivity and consistency, young children benefit from simple, direct, and developmentally appropriate explanations of experience. Age‐appropriate information about negative life events, provided in the context of a supportive relationship, affords even young children the opportunity to explore feelings of sadness, loss, loneliness, anger, and guilt, and provides a relational base for communication and a sense of safety, protection, and security.

In addition to the importance of direct caregiving experience, the relationships between alternative caregivers and incarcerated mothers have important implications for child adjustment. Research with children of incarcerated parents—although not mothers specifically—has found that alternative caregivers frequently serve as a gatekeeper between the child and the incarcerated parent, determining whether and how contact (e.g., phone, mail, visits) occurs (Shlafer & Poehlmann, 2010). Although caregivers may limit contact with good intention (e.g., to minimize children's distress following communication with the parent), most children still want and need to see their parents. Further, maintaining and supporting the mother–child relationship during the mother's incarceration in a safe and supportive manner may impact the child's overall adjustment and postincarceration transition.

#### 1.7.2 Parent–child contact

Parent–child contact (both the amount and quality) during a parent's incarceration is a key issue for children, incarcerated parents, alternative caregivers, and the professionals who serve them (Poehlmann, Dallaire, Loper, & Shear, 2010). Although the majority of incarcerated parents have some contact with their children while serving a sentence, in general, mail contact is more common than visitation (Maruschak et al., 2010). Fewer than half of state prisoners report receiving a visit with their children during incarceration (Glaze & Maruschak, 2008). As noted previously, parent–child direct contact (i.e., visits) are often limited by the distant location of the correctional facility, the high cost of transportation and long‐distance telephone calls, and the visiting environment (Poehlmann et al., 2010). Indeed, more than 60% of parents in state prisons are incarcerated more than 100 miles from their last place of residence.

The quality of children's visits with their incarcerated parents is likely affected by institutional conditions (Shlafer, Loper, & Schillmoeller, 2015) that may vary from child‐friendly (e.g., developmentally appropriate toys and family activities) to highly stressful (e.g., strict rules about children's behavior, no physical contact such as hugging or hand‐holding). Research on the effects of visitation on child outcomes has been mixed, although to date, only one study has included systematically observed child behavior in the context of visits, and this work was conducted with fathers in jail (Poehlmann‐Tynan, Burnson, Runion, & Weymouth, 2017).

### 1.8 After incarceration

There has been considerable research on the barriers to formerly incarcerated individuals’ reentry into their communities; yet, little is known about how postincarceration experience impacts children. Economic stability, employment, housing, and social support are among the documented challenges that returning citizens face postincarceration, and each has implications for child well‐being. For example, a mother or father's incarceration often results in the loss of household income. Among parents who lived with their minor children in the month before their arrest or just prior to their incarceration, nearly all mothers (89%) and most fathers (67%) report providing financial support to the family (Glaze & Maruschak, 2008). This loss of financial support may impact the family's housing stability, the child's living arrangement, and continuity in childcare experience. When released from prison, parents face numerous barriers to regaining employment and housing. Providing financial support to one's child and family may be a priority, but finding gainful employment or affordable housing postincarceration may be challenging.

There has been limited research examining how parents navigate relationships with their children and families upon release. In their study with 38 women who were in prison in Arkansas, Harm and Phillips (2001) described family “as both the best and most difficult part of returning to the community” (p. 10). As Harm and Phillips described, for mothers following release from prison, their relationships with their children may be both a source of support (e.g., companionship) and a source of stress (e.g., fear about not being able to provide for children). Indeed, other scholars have written about parenting stress as a gender‐specific risk factor for recidivism among women (e.g., Van Voorhis, Wright, Salisbury, & Bauman, 2010).

For some parents, particularly mothers, who plan to return to providing care for their children, this transition can often be a source of stress and conflict. The transition requires family members to renegotiate roles and responsibilities, which may also be difficult and confusing for young children. When parental incarceration improves aspects of the caregiving environment (e.g., removing a physically abusive parent from the home), parental release from prison can result in considerable fear or stress (e.g., about the family's safety, undesired parent–child contact). More research is needed to better understand the parental reentry process (within family systems as well as communities) following incarceration and its impact on young children.

#### 1.8.1 Summary

National estimates from the Bureau of Justice Statistics indicate that approximately 1.75 million children had a parent in state or federal prison in the United States in 2007 (Maruschak et al., 2010). More recent research has indicated that more than 5 million U.S. children have had at least one incarcerated parent (Murphey & Cooper, 2015). The incarceration of a parent encompasses a sequence of events and transitions that exposes young children to a range of risk factors that may compromise health and development across the life course. Although incarceration is likely not *the cause* of such compromised outcomes, combined with other co‐occurring risks and vulnerabilities, parenteral incarceration make families particularly fragile. Given the potential long‐term consequences of parental incarceration for child and adult health, there is a significant need for targeted, evidence‐informed prevention and intervention efforts and related policies.

## 2 IMPLICATIONS FOR POLICY AND PRACTICE

### 2.1 Policy and practice: Reproductive heath

With the recent dramatic rise in female incarceration, there is an urgent need to update correctional policies and practices to address the unique vulnerability and health needs of incarcerated women and their young children. Consistent with a reproductive justice framework, this includes making concerted efforts to provide incarcerated women with comprehensive health services before, during, and after pregnancy, and for those who decide to have a baby, with support during labor, delivery, and the postpartum period (e.g., breast‐feeding opportunities). In addition, practices are needed to promote the healthy development of relationships between incarcerated mothers and their infants and young children. To support these goals, policy development must include attention to (a) the systematic identification of pregnant women (i.e., routinely offering pregnancy testing); (b) limiting or banning the use of restraints on incarcerated women during pregnancy, labor, and delivery, and the postpartum period (ACLU, 2012; American College of Obstetrics and Gynecologist, 2011, 2012); (c) providing adequate prenatal care (e.g., medical examinations, screening, and treatment for high‐risk pregnancies), nutrition, and activity levels for pregnant incarcerated women (e.g., Shlafer, Stang, Dallaire, Forestell, & Hellerstedt, 2017); (d) supporting opportunities for mother–infant bonding and attachment during the postpartum period and beyond, including supporting the mother's right to initiate breast‐feeding (e.g., Kotlar et al., 2015) and having consistent, child‐friendly contact visitation; and (e) increasing opportunities for mothers to participate in community‐based alternatives to incarceration (e.g., day programs or residential programs), which would prevent the separation of mothers from their infants, and increase opportunities for mother–infant bonding, attachment, and breast‐feeding (Women's Prison Association, 2009).

In addition to direct health services and practices, auxiliary support is likely critical for incarcerated women who often experience stressful and high‐risk pregnancies (Barkauskas, Low, & Pimlott, 2002), which may increase one's risk for maternal depression, preterm birth, and low‐birth‐weight infants. Supportive services may include (a) health education regarding pregnancy, childbirth, and parenting; (b) opportunities for contact visitation with family and friends; (c) doulas and midwives before, during, and after delivery (Barkauskas et al., 2002); and (d) community‐based support for mothers and their families when women are released from prison or jail.

Many states and the federal government have introduced or enacted legislation aimed at improving the reproductive health of incarcerated women, including limiting the use of restraints and improving access to comprehensive reproductive healthcare (American Psychological Association, 2017). We encourage all states and the federal government to enact policies that consider alternatives to incarceration for pregnant women. When alternatives are not available, policies should ensure access to comprehensive prenatal care and postpartum support that promote the health of incarcerated women and their infants and young children.

One approach to promoting reproductive justice among incarcerated pregnant women is doula childbirth assistance and support. Doula programs are available for pregnant and laboring women in several corrections facilities across the United States, with preliminary, but promising, results related to health indicators and maternal satisfaction (e.g., Kotlar et al., 2015; Schroeder & Bell, 2005; Shlafer, Gerrity, Ruhland, & Wheeler, 2013). As an example, the Minnesota Prison Doula Project (Shlafer et al., 2013) utilizes trained doulas who provide group‐based and individual education and support for new mothers in a state prison with the goal of nurturing healthy mother–child relationships and increasing parenting confidence. In pilot data, participants ranged in age from 18 to 40 years, and represented diverse rural and urban communities from around the state. Few participants experienced their first pregnancies while incarcerated whereas others did not know that they were pregnant prior to arriving at the prison. Participants’ satisfaction with the doula program was rated as high, and pilot data have demonstrated promising results in terms of birth outcomes, with very low rates of cesarean deliveries, and few preterm or low‐birth‐weight infants (Shlafer et al., 2013).

Despite promising efforts, there remains a great need for evidence‐based programming for incarcerated pregnant women to promote individual maternal health and development as well as family rebuilding and reunification (Dallaire & Shlafer, 2017). Research would benefit from multisite designs (to increase sample size), the use of observational measures in addition to self‐report tools, and the alignment of outcome assessments with programmatic goals. Further, strong university–corrections–community partnerships may facilitate program delivery and evaluation (Shlafer, Gerrity, & Duwe, 2015).

### 2.2 Policy and practice: Infants and young children with incarcerated mothers

Preserving and strengthening the relationship between child and parent while a parent is incarcerated promotes permanency and reduces the potentially damaging effects of separation (Klain et al., 2009). The Second Chance Act (H.R. 1593, 110th Cong., § 243, 2008; https://www.congress.gov/bill/110th-congress/house-bill/1593/text) supports state, local, and tribal governments and nonprofit organizations in their work to reduce recidivism and improve outcomes for individuals returning from state and federal prisons, local jails, and juvenile facilities. The Act calls for the development of best practices for

communication and coordination between such State departments and agencies to ensure the safety and support of children of incarcerated parents (including those in foster care and kinship care), and the support of parent‐child relationships between incarcerated (and formerly incarcerated) parents and their children, as appropriate to the health and well‐being of the children. (p. 122)

Several programs and practices aimed at maintaining parent–child relationships during parental incarceration exist, although published program evaluations are rare. We describe examples of these efforts next.

#### 2.2.1 Community‐based alternatives to incarceration

Community‐based residential programs that offer alternatives to incarceration, while also providing parenting education, supportive housing, and substance‐abuse counseling, provide promising approaches (for a review, see Women's Prison Association, 2009). Community programming supports the development of mother–infant relationships and aims to prevent children from entering foster care, with positive effects on relapse and recidivism. In her ethnographic study of one program which provided supportive housing and wraparound services as an alternative to incarceration, Goshin (2015) described the experiences of formerly incarcerated women who lived in the unit, their children, program staff, program administrators, and prosecutors. Participants described the program as providing women with a safe space to live, allowing them the opportunity to complete court requirements, while being able to reside with and provide care for their children.

In their study of an experimental, community‐based, residential program for pregnant women with short‐term sentences, Barkauskas et al. (2002) found that program participants had similar pregnancy and delivery outcomes relative to the comparison group, but that program participants were significantly more likely to be breast‐feeding their infants at hospital discharge. They also noted low rates of positive drug screens for program participants and concluded that “a structured, supportive environment can protect the health of the fetus while also promoting maternal–newborn attachment and positive maternal health” (p. 378). Community‐based alternatives to incarceration offer a promising approach to reduce the number of women who are incarcerated while also providing critical opportunities for mothers to remain with their children. Further research on these policy alternatives and their implications for maternal and child health is warranted.

#### 2.2.2 Child‐friendly visitation

Innovative programs (e.g., “Get on the Bus,” California Department of Corrections and Rehabilitation; https://www.cdcr.ca.gov/visitors/visitors/get-on-the-bus/) have attempted to reduce barriers to in‐person parent–child visits by providing free transportation for children and their alternative caregivers to visit incarcerated parents. In‐person visits that provide mothers with an opportunity to hold and directly interact with their infants and young children are developmentally appropriate, and when in the best interests of the child, opportunities for court‐approved contact support the child–parent relationship during and following parental incarceration.

Child welfare agencies are responsible for services, including visitation, that promote the reunification of families. In the absence of reunification goals, however, planned contact or acknowledgement of the parent–child relationship and its meaning for a particular child is important for the emotional well‐being of young children. Advocacy for visitation guidelines that promote and maintain healthy relationships for young children is needed. Guidelines must include the availability of developmentally appropriate and child‐friendly visitation environments, opportunities for direct physical contact between the child and parent, and monitoring of the child's experience and needs surrounding visitation.

When in‐person contact is not advisable or possible, other options for keeping a parent alive in a child's mind include the use of supportive video visitation (e.g., Osborne Association, New York, NY, http://www.osborneny.org/). Other innovative programs include supporting parents with telephone contact and exchanging personal photographs, letters, and audio recordings of story reading (e.g., Read to Me, Minneapolis, MN, https://www.hclib.org/about/outreach). The programs and policies for supporting child‐friendly visits vary from state to state (Shlafer, Loper, & Schillmoeller, 2015), but recent initiatives funded through the National Institute of Corrections (2017) have begun to identify promising approaches to supporting healthy parent–child contact when parents are incarcerated.

#### 2.2.3 Family‐based support services

In the context of parental (especially maternal) incarceration, alternative caregivers play a significant role of the child's experience, often as a gatekeeper between the child and incarcerated parent. Innovative approaches to overcoming this barrier include models such as *Look Up and Hope* (Ryba, Gibertson, & Meyerson, 2012). With complex family dynamics in mind, the *Look Up and Hope* initiative works with the family unit (incarcerated mother, minor children, and alternative caregivers) to provide comprehensive services (e.g., educational and employment services, family‐centered mental health assessment and treatment, parenting classes, graduated visitation opportunities) in an effort to improve relations within families affected by maternal incarceration. In evaluation, 80% of *Look Up and Hope* families experienced improved family relationships (i.e., increased contact and successful reunification with family members) as well as improved parenting skills, support for child educational activities, and chemical health status (Ryba et al., 2012).

Auxiliary supports to improve communication among caregivers include exemplary templates for letters from incarcerated mothers to alternative caregivers that focus on child needs and the importance of creating a shared understanding of the child's experience. Additional examples of useful resources focusing on practices that connect children with incarcerated parents include:

- *Visiting Mom or Dad: The Child's Perspective* (Adalist‐Estrin, 2003), a fact sheet for caregivers and child advocates offers practical tips on preparing children for prison visits with developmental guides for different‐aged children.
- The *Child Protection Best Practices Bulletin* (Corinne Wolfe Children's Law Center, Advocacy, Inc., New Mexico CASA Network, University of New Mexico, New Mexico Children, Youth, and Families Department, New Mexico Citizens Review Board, & New Mexico Children's Court Improvement Commission, 2011) reviews the advantages of promoting and maintaining these child parent connections, including allowing the child to develop a more realistic understanding of the parent location and circumstances, to know the parent is safe, to express emotional reactions to separation from the parent, and to maintain relationships that may contribute to successful family reunification.
- The *New York Initiative for Children of Incarcerated Parents*, *Stronger Together Handbooks* (Brooks, Gaynes, Krupat, Lemaster‐Schipani, et al., 2013; Gaynes, Krupat, Lemaster‐Schipani, & Hunt, 2013; Wallace, Glaser, Rafael, Baniak, Krupat, et al., 2013) address experiences of children of incarcerated parents, the importance and methods of maintaining relationships between children and parents, and the role of alternative caregivers.
- Sesame Street Workshop's *Little Children, Big Challenges: Incarceration* toolkit (Sesame Street, 2013) provides a range of resources for service providers, parents, and families, including tips for incarcerated parents, activities, and videos.

## 3 RESEARCH, PUBLIC EDUCATION, AND ADVOCACY

Protecting and supporting the reproductive health and healthy development of infants and young children with incarcerated mothers requires visibility. Visibility and increased understanding of the unique needs of women in prison and the experiences of infants and young children separated from their mothers derive from systematic record keeping and research, public education and conversation, and advocacy for policy and practice change.

### 3.1 Data collection and research

Currently, many jurisdictions across the country do not uniformly keep records regarding parents and pregnant women who are incarcerated and children of adults who come in contact with the criminal justice system. Both criminal justice and child welfare systems can contribute to increasing the visibility of incarcerated parents and children of incarcerated parents by routinely requesting and collecting family information when an adult is arrested. Simple relevant statistics include the number of mothers (and fathers) incarcerated and the number and age of children with incarcerated parents by state or county. In addition to the prevalence of parental incarceration and related minor children, comprehensive data collection might address child living arrangements, relationships with caregivers, and direct experiences of parent arrest and of reunification (e.g., see the *Mothers Behind Bars* report, National Women's Law Center, 2010). In addition, cross‐agency data integration (e.g., corrections and human services) would provide opportunities for research aimed at understanding child and family outcomes when parents are incarcerated and providing essential information to guide intervention development.

### 3.2 Public education

Advocacy efforts focus on education of providers, children, and families as well as the general public about experiences of incarcerated mothers and their children. These educational efforts aim to address common misconceptions and biases about parents who are incarcerated and the perceived best interests of the child of an incarcerated parent. Common child‐related assumptions include ideas that young children fare better not knowing or seeing a parent who is in jail or prison and that young children are naturally resilient in the face of trauma or separation from a parent. Useful educational materials include a children's Bill of Rights (San Francisco Partnership of Incarcerated Parents, 2005) and the following media resources:

- https://www.theatlantic.com/education/archive/2015/11/how-parental-incarceration-affects-a-childs-education/414720/
- https://www.theatlantic.com/education/archive/2017/01/how-mass-incarceration-pushes-black-children-further-behind-in-school/513161/
- https://well.blogs.nytimes.com/2016/04/26/when-parents-are-in-prison-children-suffer/?_r=0

### 3.3 Advocacy in practice

In addition to public education, representatives of the legal (attorneys, judges, guardians ad litem), child welfare, and public health systems who work with children with parents involved in the criminal justice system may serve as important educators and agents for change, advocating for visitation of infants and young children with parents that ensure child safety and well‐being. Child attorneys and judges are in positions to propose or facilitate case plans that include safe and healthy child–parent contact or visitation in the least restrictive environment possible. As professionals responsible for interviewing children and caregivers with specific knowledge of child circumstances and specific needs, guardians ad litem may advocate for the child based on individualized information. Parent attorneys may also have opportunities to educate parents about their rights, responsibilities, possible alternatives to incarceration, and options for maintaining contact with children during incarceration. Evidence‐based child welfare practices that are child‐ and trauma‐informed can ameliorate the stress that children may experience surrounding the parent arrest and incarceration. Finally, trained infant mental health professionals can be useful partners in creating safe and therapeutic experiences for young children.

Overall, programming must be systematically evaluated to document parent (and provider) experiences and to develop a body of research that can be shared among professionals, with the goal of developing evidence‐informed practices to support incarcerated parents and public health programs to prevent involvement in the criminal justice system. Documenting and evaluating adaptations to existing evidence‐based programs designed for other high‐risk populations (e.g., parents with substance‐abuse problems) are valuable areas for future inquiry. Across disciplines and regions, it will be important for researchers, practitioners, and other professionals working within the context of incarceration to collaborate to promote public education regarding the experiences of incarcerated mothers and their children and to advocate for policies, practices, and resources that promote reproductive health of incarcerated mothers and healthy developmental trajectories of young children.

### 3.4 Conclusion

Finally, all of the challenges and barriers described in this article do not happen within a vacuum. It is not lost on us that there are disproportionate numbers of women who are mothers to Black and Brown children who are incarcerated. A failure to understand and dismantle the systems that contribute to the unequal proportion of mothers of color in the criminal justice system must be placed within the context of this body of literature. Understanding the systemic inequities that are driving disparities in mass incarceration is critical to promoting justice and equity. Women of color developed the reproductive justice framework to analyze intersecting forms of oppression and to promote human rights. This framework is fundamental to the challenges of parental incarceration and child well‐being, and has particular significance for women of color who are disproportionately represented in the criminal justice system. As such, we recommend actively and meaningfully engaging currently and formerly incarcerated women of color in identifying and implementing solutions. Dismantling systemic racism, including the policies, institutions, and procedures that advantage some and disadvantage others, is vital to this work. Ultimately, we hope to see fewer people, including women and mothers of color, in the criminal justice system.
